# Temporal trends in physical activity levels across more than a decade – a national physical activity surveillance system among Norwegian children and adolescents

**DOI:** 10.1186/s12966-021-01120-z

**Published:** 2021-04-26

**Authors:** Jostein Steene-Johannessen, Sigmund Alfred Anderssen, Elin Kolle, Bjørge Herman Hansen, Mari Bratteteig, Emilie Mass Dalhaug, Lars Bo Andersen, Wenche Nystad, Ulf Ekelund, Knut Eirik Dalene

**Affiliations:** 1 Department of Sports Medicine, Norwegian School of Sport Sciences, PO Box 4014, Ullevål Stadion, 0806 Oslo, Norway; 2 Western Norway University of Applied Sciences, Department of Sport, Food and Natural Sciences, Campus Sogndal, Sogndal, Norway; 3 Department of Chronic Diseases and Ageing, Norwegian Institute of Public Health, Oslo, Norway

**Keywords:** Physical activity, Temporal trends, Accelerometer, Children, Adolescents

## Abstract

**Background:**

There is a scarcity of device measured data on temporal changes in physical activity (PA) in large population-based samples. The purpose of this study is to describe gender and age-group specific temporal trends in device measured PA between 2005, 2011 and 2018 by comparing three nationally representative samples of children and adolescents.

**Methods:**

Norwegian children and adolescents (6, 9 and 15-year-olds) were invited to participate in 2005 (only 9- and 15-year-olds), 2011 and 2018 through cluster sampling (schools primary sampling units). A combined sample of 9500 individuals participated. Physical activity was assessed by hip worn accelerometers, with PA indices including overall PA (counts per minute), moderate-to-vigorous intensity PA (MVPA), and PA guideline adherence (achieving on average ≥ 60 min/day of moderate-to-vigorous PA). Random-effects linear regressions and logistic regressions adjusted for school-level clusters were used to analyse temporal trends.

**Findings:**

In total, 8186 of the participating children and adolescents provided valid PA data. Proportions of sufficiently active 6-year-olds were almost identical in 2011 and 2018; boys 95% (95% CI: 92, 97) and 94% (95%CI: 92, 96) and girls 86% (95% CI: 83, 90) and 86% (95% CI: 82, 90). Proportions of sufficiently active 15-year-olds in 2005 and 2018 were 52% (95% CI: 46, 59) and 55% (95% CI: 48, 62) in boys, and 48% (95% CI: 42, 55) and 44% (95% CI: 37, 51) in girls, respectively, resulting from small differences in min/day of MVPA. Among 9-year-old boys and girls, proportions of sufficiently active declined between 2005 and 2018, from 90% (95% CI: 87, 93) to 84% (95% CI: 80, 87)) and 74% (95% CI: 69, 79) to 68% (95% CI: 64, 72), respectively. This resulted from 9.7 min/day less MVPA in boys (95% CI: − 14.8, − 4.7; *p* < 0.001) and 3.2 min/day less MVPA (95% CI: − 7.0, 0.7; *p* = 0.106) in girls.

**Conclusions:**

PA levels have been fairly stable between 2005, 2011 and 2018 in Norwegian youth. However, the declining PA level among 9-year-old boys and the low proportion of 15-year-olds sufficiently active is concerning. To evaluate the effect of, and plan for new, PA promoting strategies, it is important to ensure more frequent, systematic, device-based monitoring of population-levels of PA.

**Supplementary Information:**

The online version contains supplementary material available at 10.1186/s12966-021-01120-z.

## Introduction

Sufficient levels of physical activity (PA) are associated with several health benefits in children and adolescents [1, 2], and current evidence calls for children and adolescents (6–17 years) to do an average of ≥60 min of daily moderate­to­vigorous intensity PA (MVPA) in order to achieve these benefits [3]. A number of studies published over the last 20 years have concluded that a large proportion of young people are insufficiently physically active [4–6]. Available evidence on temporal trends from large scale studies do not, however, indicate that PA levels have changed much since the 1980s [5, 7]. Therefore, it has been a global priority to decrease the prevalence of physical inactivity by 10% in 2025 [8], later by 15% in 2030 [9]. However, most temporal trend estimates are based on self-reported PA data. This is an important limitation as recall- and social desirability biases may introduce well known flaws in the interpretation of prevalence estimates [10, 11], especially in children and adolescents [11].

Device-based assessments of PA overcome several of the challenges related to self-report methods. So far, large population- and device-based PA data from children and adolescents are limited to a few countries [12–17], and studies reporting on temporal changes in PA are even fewer [14–17]. Device-based PA data from children and adolescents from the National Health and Nutrition Examination Survey (NHANES) revealed an increase in overall PA from 2003–2004 to 2005–2006 for children, but not adolescents, without any changes observed for time spent in MVPA [14] . Similarly, observations from a large, population based Canadian study revealed that MVPA levels among children and youth were stable between 2007 and 2015 [15]. In contrast, another population based study of Canadian children and youth comparing pedometer assessed PA showed a decrease in steps/day during the same time period (2005–2014) [17]. Additional evidence from smaller, non-nationally representative studies of temporal PA trends [18–24], is inconclusive. Thus, there is a critical need for device-based data on temporal trends from large scale, representative samples to study the effect of efforts made to increase PA levels. Based on existing literature [4, 6], gender and age are among the most important predictors of PA levels during childhood and adolescents. Thus, it is of great importance to explore both gender and age-related temporal trends in order to provide as comprehensive information toward preventive strategies as possible. In addition, exploring potential trends in PA during different day segments (morning, school hours, afterschool and afternoon), between weekday and weekend days, and between the tails of the PA level distributions (i.e. the least and most active) could provide even more detailed information for planning of preventive efforts.

In Norway, a national surveillance system for device measured PA in children – the Physical Activity in Norwegian Children Study (PANCS), was initiated in 2005. Since then, three surveillance studies [25–27] have been conducted (2005, 2011 and 2018), providing a unique opportunity to examine temporal PA trends and thus provide indispensable knowledge in order to carry out targeted actions and interventions, and to guide global and national planning of policy action towards ensuring that the population is achieving sufficient levels of PA.

The aim of this study was to describe gender and age-group specific temporal trends in device measured PA between three timepoints in large samples of children and adolescents from all over Norway. In secondary analyses, we further explore temporal changes separately for weekdays, weekend days and during different school day segments (morning, school hours, afterschool and afternoon), and separately for the 20% least and 20% most active individuals.

## Methods

### Participants and recruitment

**Fig. 1:**
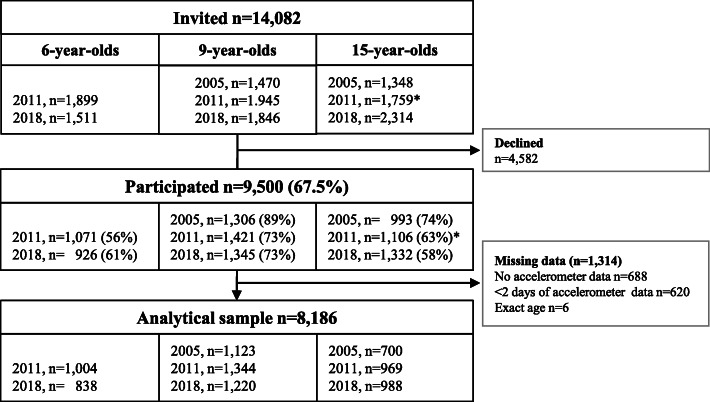
Number of participants that were invited, participated, provided valid PA data and were included in the main analyses of the present study by PANCS cohort and age group. *1119 of the invited had previously participated in 2005 when aged 9, of which 731 chose to participate when aged 15 years in 2011

An overview of recruitment, participation and valid measures from the different PANCS cohorts are given in Fig. 1. The participating children and adolescents were surveyed in 2005, 2011 and 2018. In total, 9500 (51.1% boys) of the 14,082 invited agreed to participate, yielding an overall participation rate of 67.5%. Participation rates ranged from 56.4% in 6-year-olds in 2011 to 88.8% in 9-year-olds in 2005. From the 9500 participants, 8186 had sufficient data and were included in the analytical sample (Fig. 1). Numbers and proportions of boys and girls in the analytical sample are displayed by cohort and age group in additional file 1. The recruitment procedures and methods used in PANCS1 and PANCS2 have been described in detail elsewhere [16, 25]. Briefly, in PANCS1 Statistics Norway selected nationally representative samples of 9- and 15-year-olds using cluster sampling with schools as the primary unit. PANCS2 had a mixed design; Statistics Norway selected new nationally representative samples of 6- and 9-year-olds, whereas 15-year-olds were invited either individually based on previous participations in PANCS1 or selected from a random sample of the lower secondary schools that had previously participated in PANCS1. In PANCS3, 1st, 4th and 10th graders from the same schools that participated in PANCS1 (10th grade) and PANCS2 (1st and 4th grade) were invited. If a school declined to participate, we invited another school from the same, or a corresponding, geographical and socio-demographic area. When schools agreed to participate based upon our invitation, we invited all 1st and/or 4th and/or 10th grade pupils. All participants had a physical examination at their school. Trained investigators took all measures, and identical study protocols were used in all three study waves.

All studies were carried out in accordance with the declaration of Helsinki. The Regional Committee for Medical Research Ethics (RCMRE) approved PANCS1. PANCS2 and 3 were considered outside the Health Research Act’s scope by the RCMRE and was therefore not considered subject to approval. The Norwegian Social Science Data Services AS approved all three studies. A signed informed consent from participants and their parents/legal guardians was collected before the start of the data collections.

### Demographics and anthropometry

We used the participants parent/legal guardian with the highest attained education level as a proxy for socioeconomic status, categorized as ‘low’ (primary school or lower secondary school), medium (high school (vocational or general studies)), and high (University College or University degree). In PANCS1 and 3, parents self-reported highest attained education level, whereas registry data on parental education was provided by Statistics Norway in PANCS2. We measured height to 0.1 cm (wall mounted measuring tape or Seca 899 stadiometer (SECA GmbH, Hamburg, Germany), weight to 0.1 kg (SECA 770 and 877 scales (SECA GmbH, Hamburg, Germany)) and waist circumference (WC) to 0.1 cm (measuring tape at the minimum circumference between the lowest rib and the iliac crest), and calculated body mass index (BMI) using the standard formula (kg/m^− 2^)). The WC measurements were performed twice, and the average of the two measurements recorded. All participants wore light clothing and no shoes during the anthropometric examination.

### Physical activity level, and PA guideline adherence

We assessed PA using ActiGraph accelerometers (ActiGraph, LLC, Pensacola, Florida, USA), which participants wore on their right hip. In PANCS1, we used the CSA 7164 model and instructed the participants to wear the monitor for all waking hours (except during showering and bathing) for five consecutive days, including two weekend days. In PANCS2 and 3, we used the GT1M and GT3X+ models and instructed the participants to wear the monitor during all waking hours (except during showering and bathing) for eight consecutive days. We initialized the monitors to start recording at 06:00 the day after the participants received them, yielding a per protocol maximum of 4 and 7 days of recording in PANCS1 and PANCS2/3, respectively. We used the ActiGraph RIU software in PANCS1 (K64, Computer Science & Application Inc., Shalimar, Florida, USA) and the ActiLife software in PANCS2/3 (ActiGraph, LLC, Pensacola, Florida, USA) to initialize the monitors and to download the accelerometer files. For further processing (vertical accelerations only), we used KineSoft (KineSoft version 3.3.80, Loughborough, UK) and Stata (StataCorp. 2013. Stata Statistical Software: Release 13. College Station, TX: StataCorp LP.).

Due to the sporadic nature of children’s PA, an epoch period of 10 s was used. After excluding data recorded from 00:00 to 06:00 and all intervals of ≥20 consecutive minutes with no counts recorded (defined as non-wear), we considered days with ≥480 min of activity recordings valid. We chose to include all participants with ≥2 days of valid activity recordings for our main analyses, yielding an analytical sample where 99.8% had ≥1 valid weekday and 87.2% had ≥1 valid weekend day. This combination of non- wear, valid day, and number of valid days criteria has previously been shown to give reliability coefficients of > 80% in both children with (87%) and without (81%) valid weekend data [28]. For our secondary analyses of weekdays, weekend days, and different school day segments (morning, school hours, afterschool and afternoon), we applied the inclusion criteria presented in additional file 2.

We used average counts min^− 1^ (CPM), calculated by dividing the total number of counts by the total number of valid wear minutes, as a measure of overall PA. To estimate time spent in MVPA, we used the widely applied European Youth Heart Study cut-point of ≥2000 CPM, which corresponds to a walking speed of approximately ≥4 km/h in young people [29]. Participants achieving on average ≥ 60 min/day of MVPA were defined as being sufficiently physically active.

In PANCS1 and 3, we collected accelerometer data during all months of the year except in July and August. In PANCS2, we collected accelerometer data from March to December, apart from July.

### Statistics

Descriptive characteristics are presented as means and standard deviations (SD), unless otherwise stated. Differences in background characteristics between cohorts are analysed using random effects linear regression adjusted for school-level sampling (see below). Means and percentages ​​of the dependent variables (CPM, MVPA, and PA guideline adherence), with their corresponding standard errors / confidence intervals (SE / 95% CI), are predicted using Statas margins command following each temporal trend analysis (adjusted for covariate differences between cohorts and school-level sampling). Temporal trends in PA were analysed using random-effects linear regression adjusted for potential intragroup correlations from school-level (cluster) sampling. Logistic regression (also adjusted for the school-level sampling) was used in analyses comparing proportions adhering to the PA guidelines. To account for potential differences between the cohorts that may impact PA other than time, all analyses were adjusted for age and seasonality (minutes of daylight) at the time of PA assessment. In analyses of MVPA and PA guideline adherence, models were additionally adjusted for accelerometer wear time. For the secondary analyses comparing “the most and least active” participants between cohorts (in terms of daily MVPA), we created cohort, age and gender specific quintiles and repeated temporal change analyses for MVPA and PA guideline adherence for the 20% most and 20% least active participants. All statistical analyses were performed in Stata SE 13.1 (StataCorp. 2013. Stata Statistical Software: Release 13. College Station, TX: StataCorp LP).

## Results

In total, 1842 (92.2%) of the participating 6-year-olds, 3687 (90.5%) of the participating 9-year-old and 2657 (77.4%) of the participating 15-year-olds provided valid PA data and were included in the main analyses (Fig. 1). Table 1 displays background characteristics of the study sample by age, gender, and cohort suggesting slightly lower BMI or WC levels in 2011 and/or 2018 compared to 2005 in all age-groups. The 15-year-old girls and boys in the 2011 and 2018 cohorts were also significantly younger than their peers in the 2005 cohort.

**Table 1:** Descriptive characteristics (mean (SD)) of participants includeded in the main analyses of temporal trends in physical activity (i.e.with ≥2 days of valid of accelerometer data) by agegroup, cohort and sex (*n* = 8186)^a^

	n ^**a**^	Age (yrs.)	Height (cm)	Weight (kg)	BMI (kg.m^**−2**^)	WC (cm)	Socioeconomic status ^**b**^			
							Low (%)	Medium (%)	High (%)	Missing (%)
**BOYS**										
6-y-olds										
2011	494 (475)	6.6 (0.4)	122.2 (5.8)	24.0 (3.7)	16.0 (1.6)	54.8 (4.2)	4.5%	33.2%	61.1%	1.2%
2018	411 (403)	6.5 (0.4)	121.5 (5.5)	23.4 (3.6)	15.8 (1.6)*	54.6 (4.0)	2.9%	20.9%	60.1%	16.1%
9-y-olds										
2005	599 (593)	9.6 (0.4)	139.9 (6.2)	33.9 (6.3)	17.2 (2.4)	62.1 (7.2)	9.7%	34.1%	44.1%	12.2%
2011	652 (630)	9.6 (0.4)	138.7 (6.8)	34.0 (7.0)	17.6 (2.8)	60.8 (6.7)*	5.8%	39.1%	53.4%	1.7%
2018	607 (597)	9.5 (0.4)	139.2 (6.3)	33.9 (6.5)	17.4 (2.5)	60.9 (6.2)	1.2%	17.0%	61.8%	20.1%
15-y-olds										
2005	340 (334)	15.6 (0.4)	175.6 (7.1)	65.2 (12.8)	21.1 (3.7)	75.5 (9.4)	5.6%	32.9%	34.7%	26.8%
2011	480 (430)	15.2 (0.6)*	173.2 (8.0)*	62.3 (12.0)*	20.7 (3.3)	73.1 (8.7)*	7.3%	38.1%	50.4%	4.2%
2018	491 (444)	15.4 (0.4)**	175.1 (7.4)**	63.5 (11.3)	20.6 (3.0)	72.7 (6.9)*	2.9%	15.1%	56.4%	25.7%
**GIRLS**										
6-y-olds										
2011	510 (493)	6.6 (0.4)	120.9 (5.4)	23.8 (4.2)	16.2 (2.0)	54.4 (5.0)	5.3%	32.2%	60.6%	2.0%
2018	427 (412)	6.5 (0.4)	120.7 (5.3)	23.3 (3.6)	15.9 (1.7)**	53.8 (4.3)	2.3%	15.9%	64.4%	17.3%
9-y-olds										
2005	524 (518)	9.6 (0.4)	138.4 (6.8)	34.0 (7.1)	17.6 (2.7)	63.3 (7.7)	6.7%	38.4%	45.0%	9.9%
2011	692 (679)	9.6 (0.4)	138.0 (6.5)	33.7 (6.8)	17.6 (2.7)	59.4 (6.5)*	7.4%	35.3%	54.9%	2.5%
2018	613 (594)	9.5 (0.4)	138.3 (6.5)	33.8 (7.0)	17.5 (2.6)	59.6 (6.6)*	2.6%	20.7%	57.1%	19.6%.
15-y-olds										
2005	360 (356)	15.6 (0.4)	165.6 (6.5)	58.0 (8.7)	21.1 (2.8)	73.3 (6.9)	8.3%	36.9%	34.7%	20.0%
2011	489 (422)	15.2 (0.6)*	165.0 (6.2)	57.4 (9.5)	21.1 (3.1)	69.2 (6.7)*	6.1%	38.2%	53.0%	2.7%
2018	497 (457)	15.4 (0.4)**	165.4 (6.1)	58.3 (9.9)	21.3 (3.3)	68.8 (6.9)*	3.6%	13.3%	58.8%	24.4%

*Abbreviations: yrs.* years, *BMI* body mass index, *WC* waist circumference

* Significantly different from 2005 (from 2011 in 6-y-olds) (*p* ≤ 0.049)

** Significantly different from 2005 & 2011 (*p* < 0.01)

^a^ n varies for the different anthropometric measurements and is lowest (in parenthesis) for WC except in 15-y-old boys (weight) and 15-y-old girls (BMI)

^b^ Based on parental education level (the parent with the highest attained education level), with low = primary school or lower secondary school, middle = high school (vocational or general studies), and high = University College or University degree. Note that parental education levels are based on self-reports in 2005 and 2018, and from linkage with registry data (Statistics Norway) in 2011

### Temporal trends in PA

**Fig. 2:**
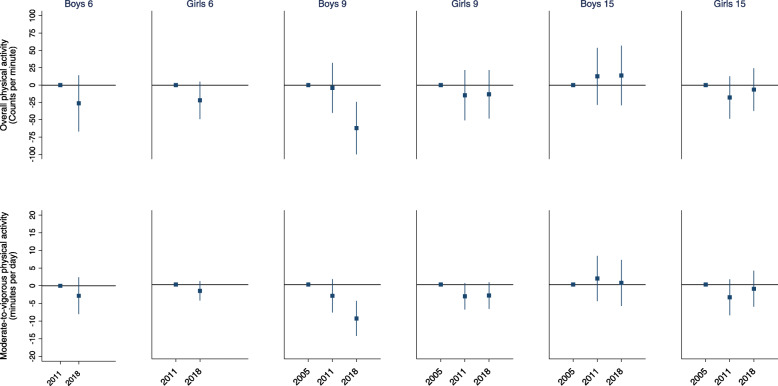
Temporal changes in overall physical activity (CPM) and daily minutes of moderate-to-vigorous physical activity between 2005, 2011 and 2018 in 6-, 9- and 15-year-old boys and girls. n varies from 411 (boys in 2018) to 510 (girls in 2011) among 6-year-olds, from 524 (girls in 2005) to 692 (girls in 2011) among 9-year-olds and from 340 (boys in 2005) to 497 (girls in 2018) among 15-year-olds. Error bars are 95% confidence intervals

Figure 2 (and Table 2) shows temporal changes in overall PA and daily minutes of MVPA between 2005, 2011 and 2018. In general, we observed only small changes in overall PA between 2005, 2011 and 2018. The exception is found in 9-year-old boys, where we observe a significantly lower overall PA level in 2018 compared to 2005 (mean difference: 62 CPM (95% CI: 24, 100)) and 2011 (mean difference: 58 CPM (95% CI: 24, 100)). This corresponds to approximately a 10% lower overall PA level in 2018 compared to 2005 and 2011. Although findings are not significant, Fig. 2 also indicates a trend towards lower CPM over time in 6-year-olds and 9-year-old girls.

**Table 2:** Overall physical activity and time spent in MVPA weekly, during weekdays and during weekend days, and time spent in MVPA during school day segments, in 2005, 2011 and 2018^a^ All values

		Overall physical activity level (CPM)			Moderate to vigorous physical activity (minutes per day)						
		Day types			Day types			School day segments			
	n^**b**^	Weekly	Weekdays	Weekend days	Weekly	Weekdays	Weekend days	Morning	School	After school	Afternoon
**6 y old boys**											
2011	494 (350)	794 (770, 819)	797 (771, 823)	787 (746, 828)	99 (96, 102)	104 (101, 107)	86 (82, 90)	11 (10, 11)	36 (34, 38)	29 (27, 31)	32 (31, 34)
2018	411 (248)	768 (735, 801)	776 (741, 812)	728 (681, 774)#	96 (92, 101)	101 (96, 105)	81 (76, 87)	11 (10, 12)	35 (34, 37)	29 (27, 31)	29 (27, 31)*
**6 y old girls**											
2011	510 (333)	712 (692, 732)	712 (694, 730)	702 (666, 737)	82 (80, 84)	85 (83, 87)	71 (68, 74)	9 (8, 10)	30 (29, 31)	23 (22, 24)	27 (26, 28)
2018	427 (266)	690 (670, 711)	702 (679, 725)	655 (624, 685)**	80 (78, 82)	85 (82, 87)	68 (66, 71)	9 (8, 10)	29 (27, 30)	25 (23, 26)#	26 (25, 27)
**9 y old boys**											
2005	599 (395)	698 (671, 726)	742 (708, 777)	641 (603, 679)	94 (90, 98)	106 (101, 112)	78 (74, 82)	13 (12, 14)	35 (32, 38)	25 (23, 27)	38 (35, 41)
2011	652 (482)	694 (671, 717)	702 (681, 724)#	670 (632, 709)	91 (88, 94)	97 (94, 101)*	76 (72, 80)	11 (11, 12)*	33 (31, 35)	22 (21, 24)*	34 (32, 35)*
2018	607 (388)	636 (611, 662)**	653 (629, 676)**	578 (537, 619)**	84 (81, 88)**	91 (87, 94)**	69 (64, 74)**	10 (9, 11)**	31 (30, 33)#	21 (19, 22)*#	32 (30, 34)*#
**9 y old girls**											
2005	524 (328)	597 (567, 627)	615 (585, 646)	565 (524, 606)	75 (72, 78)	83 (79, 87)	65 (61, 69)	11 (10, 13)	26 (23, 28)	18 (17, 20)	31 (29, 33)
2011	692 (530)	582 (563, 602)	583 (565, 601)#	576 (540, 611)	72 (70, 74)#	76 (74, 79)*	63 (59, 66)	9 (9, 10)*	25 (23, 26)	17 (16, 18)#	27 (26, 28)*
2018	613 (405)	584 (564, 603)	594 (573, 614)	547 (518, 575)	72 (70, 74)	77 (74, 80)*	59 (55, 62)*	9 (8, 10)*	24 (22, 25)	18 (17, 19)	29 (27, 30)##
**15 y old boys**											
2005	340 (164)	479 (448, 511)	513 (477, 549)	424 (393, 455)	66 (61, 71)	76 (70, 83)	52 (44, 59)	10 (8, 12)	26 (24, 29)	13 (11, 15)	33 (29, 36)
2011	480 (260)	492 (469, 516)	507 (483, 531)	443 (408, 478)	68 (64, 71)	73 (69, 77)	52 (47, 57)	10 (9, 11)	24 (22, 26)	12 (11, 13)	32 (30, 35)
2018	491 (169)	493 (465, 522)	505 (476, 534)	444 (402, 485)	66 (62, 71)	71 (66, 75)	50 (43, 57)	10 (8, 11)	24 (21, 26)	12 (11, 13)	32 (29, 35)
**15 y old girls**											
2005	360 (201)	428 (404, 452)	448 (425, 470)	399 (364, 433)	60 (56, 64)	68 (64, 72)	47 (43, 51)	10 (9, 11)	21 (19, 24)	11 (10, 13)	29 (26, 33)
2011	489 (290)	410 (393, 428)	421 (405, 436)#	372 (344, 400)	57 (54, 60)	62 (59, 64)*	44 (41, 48)	9 (8, 10)	18 (16, 19)*	11 (10, 12)	26 (25, 28)
2018	497 (239)	422 (402, 442)	432 (412, 452)	375 (343, 408)	59 (56, 63)	64 (60, 67)	46 (41, 51)	10 (9, 11)	19 (17, 21)	12 (11, 13)	28 (25, 30)

All values are means with corresponding 95% CIs predicted using Statas margins command following each temporal trend analysis (adjusted for covariate differences between cohorts and school-level sampling)

*Significantly different from 2005 (from 2011 in 6-y-olds) (*p* < 0·05). **Significantly different from 2005 & 2011 (*p* < 0·05). # different from 2005 (*p* = 0·05–0·10). ## different from 2005 and 2011 (*p* = 0·05–0·10). *# different from 2005 (*p* < 0·05) and 2011 (*p* = 0·05–0·10)

^a^ 6-year olds did not participate in PANCS1 (2005)

^b^ n varies and is lowest (in parenthesis) for “Weekend days” except in 9- and 15-year old girls and boys in 2005 for which n is lowest for “Morning”

School day segments: Morning = 06:00–09:00; School = 09:00–13:00 (6-, and 9-y-olds)/14:00 (15-y-olds); School = 13:00 (6-, and 9—y-olds)/14:00 (15-y-olds)-16:00 = Afterschool: 16:00–00:00 = Afternoon

The same patterns are observed for MVPA. Among 9-year-old boys, lower levels of MVPA are observed from 2005 through 2011 to 2018. Mean difference from 2005 to 2011 and from 2011 to 2018 were − 3.2 min/d (95% CI: − 8.0, 1.5) and − 6.5 min/d (95% CI: − 10.9, − 4.7) respectively. Compared to 2005, 9-year-old boys thus accumulated 9.7 (95%CI: − 14.8, − 4.7; *p* < 0.001) less min/d of MVPA in 2018, corresponding to a 10.4% temporal change. Although not statistically significant, results also revealed that 9-year-old girls accumulated 3.2 min/d of MVPA less in 2018 compared with 2005 (95% CI, − 7.0, 0.7; *p* = 0.106).

### Temporal trends in the proportion of sufficiently active

**Fig. 3:**
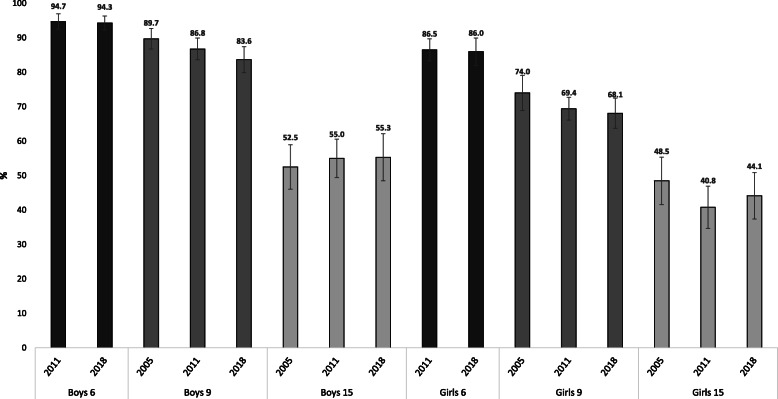
Physical activity guideline adherence (an average of 60 min/day of moderate-to-vigorous physical activity) in 2005, 2011 and 2018 in 6-, 9- and 15-year-old boys and girls. Error bars are 95% confidence intervals

Figure 3 displays the proportion of sufficiently active (≥60 min/day of MVPA) in 6-, 9- and 15-year-olds for the three PANCS cohorts. The proportion of sufficiently active 6-year-olds were almost identical in 2011 and 2018; boys 95% (95% CI: 92, 97) vs. 94% (95% CI: 92, 96) and girls 86% (95% CI: 83, 90) vs. 86% (95% CI: 82, 90). Among 9-year-olds, a reduction is observed between 2005 and 2018 in both boys (− 6.0 percentage points (95% CI: − 10.9, − 1.2)) and girls (− 5.9 percentage points (95% CI: − 12.7, 0.9)), although the reduction is only statistically significant at the 0.05-level in boys (*p*-values for boys and girls 0.014 and 0.093, respectively). In 15-year-olds, prevalence estimates do not show any clear trend and seem relatively stable at about 55% among boys and about 45% among girls.

### Day types and school day segments

In secondary analyses we also explored temporal changes during weekdays, during weekend days and during four different segments of school days (morning, school, after school and afternoon) (Table 2). For the 9-year-old boys, the weekly reductions observed between 2005 and 2018 (Fig. 2) are reflected by less accumulated minutes of MVPA across weekdays, weekend days and in all school day segments. The temporal change in MVPA from 2005 to 2018 is, however, more pronounced during weekdays than during weekend days. Temporal declines were also observed between 2005 and 2018 on both weekdays and weekend days in 9-year-old girls, with results indicating somewhat larger relative declines on weekend days (~9%) than on weekdays (~7%). Albeit not as pronounced as in 9-year-olds and with most 95% CIs including unity, Table 2 also indicates several slight, negative trends among 6-year-olds, and 15-year-olds.

### Least and most active

Additional files 3 and 4 displays temporal changes among the 20% most and the 20% least active 6-, 9- and 15-year-olds. Results indicate that 6- and 9- year-olds (boys and girls) in the most active quintile have reduced daily MVPA, ranging from a 7 min/d (6%) reduction between 2011 and 2018 in 6-year-old girls to 24 min/day (17%) reduction between 2005 and 2018 among 9-year-old boys. A similar pattern, between 5 to 11% relative reductions, is observed for the least active quintile among 6-y-olds and 9-year-old boys. No temporal change in MVPA is observed for the least active 9- and 15-year-old girls, whereas the least active 15-year-old boys reduced MVPA by approximately 4 min/d, equivalent to a 10% relative reduction. Even though reductions in MVPA are evident among the 20% most active across age groups, 100% still accumulated ≥ 60 min/d of MVPA in 2018. In contrast, the proportion of 9-year-old boys being sufficiently active decreased from 65% in 2005 to 11% in 2018 among those in the least active quintile. Similarly, the proportions among the least active 6-year-olds adhering to the PA guideline declined by ⁓20 percentage points between 2011 and 2018 (Additional file 3).

## Discussion

To the best of our knowledge, this is the largest study to explore temporal PA changes over more than a decade, using device-measured PA from population-based samples of both children and adolescents. Overall, results indicate that PA levels have remained fairly stable, but with tendencies towards decreased levels of PA between 2005 and 2018. The most pronounced change is observed in 9-year-old boys where time spent in MVPA was reduced by 10 min/d over the 13-year period. This 70 min/week average decline could for example be translated to one-two weekly fewer soccer practice sessions per week [30]. Consequently, the proportion of sufficiently active 9-year-old boys decreased by 6 percentage points from 2005 to 2018. Moreover, this temporal change seems to have occurred in both the least and most active 9-year-olds, albeit with results indicating the largest decline among the most active (Additional file 4). Although not statistically significant, our findings indicate a similar pattern towards lower PA levels over time in 6-year-olds and 9-year-old girls. Comparing temporal trends within day types and school day segments revealed that the temporal decline in PA between 2005 and 2018 in 9-year-old boys is reflected by less accumulated minutes of MVPA across day types and all school day segments. However, the change in MVPA from 2005 to 2018 is more pronounced during weekdays than during weekend days. Similarly, a non-significant negative trend of time spent in MVPA was observed in 6-year old boys and girls and in 9-year old girls during weekdays and school hours. A non-significant negative trend was also observed in 15-year-old girls during school hours between 2005 and 2011. Our findings corroborate recent pedometer-based [17] and accelerometer-based [15] data from Canada, showing stable or declining levels of steps/day and MVPA over the last 10–15 years. However, these studies only represent data from two high income countries, highlighting that more nationally representative surveys in low- and middle income countries using comparable device-based PA are warranted to strengthening the evidence base to develop a better understanding about PA trends among children and adolescents globally.

Despite launching a national PA action plan in 2004, implementation of different school-based initiatives aimed at increasing PA levels and placing the positive health benefits associated with regular PA on the political agenda, the PA levels of Norwegian children and adolescents have not increased. On the contrary – PA levels seem in decline. This might be due to several factors, among them that society’s overall effort and policy actions to increase population levels of PA have likely been insufficient. One other important distinction when discussing the lack of improvements or even decreases in PA is to take into account the potential for improvement. In a large harmonized individual participant dataset in European youth [6], Norwegian children and adolescents are among those most active and the prevalence estimates of sufficiently active children and adolescent are higher compared to many other European countries. Based on the prevalence estimates from 2018 as many as 90 and 80% among 6- and 9-year-olds, respectively, are defined as physically active. Although this could suggest limited potential for improvement, the size of the negative temporal trends and age-related declines in physical activity from childhood to adolescents observed are substantial. As there is little reason to believe that PA levels in 2005 were unsustainably high or that 60 min of MVPA/day is unattainable for 50% of 15-year-olds, a sealing effect seem unlikely. Our results therefore suggest that current policy actions aimed at increasing physical activity levels in young people have been insufficient and it is unlikely Norway will achieve the WHO global target of reducing physical inactivity by 10 and 15% in 2025 and 2030, respectively.

On the other hand, opportunities for sedentary activities seem to be ever increasing in young people. Data from the Health Behaviour in School-aged Children Study revealed that screen time increased significantly among 11-, 13-, and 15-year-olds from 2002 to 2010 in all the 30 participating countries [31]. This is in line with data from NHANES from 2001 through 2016 [32]. Moreover, recent data from a large Norwegian survey shows that time spent on digital display activities increased by 7–13% between 2015 and 2018 [33]. Thus, we could speculate that a greater uptake of screen-based technology can have attenuated the effect of initiatives put in place to increase PA, and that PA levels might have declined even further without any of these initiatives. One major problem is that the majority of national and global surveys have not assessed sedentary time spent on handheld devices, and it is thus unknown if increased screen time is a result of switching from traditional sedentary behaviours (e.g. reading and television) to more excessive use of electronic handhold devices. Nevertheless, some studies suggest only modest associations between screen time and MVPA in children and youth [26]. Even if we observed the most pronounced decline in physical activity in 9-year old boys, the consistently lower levels of physical activity in girls, suggest that future preventive efforts should address both boys and girls. Studies examining whether gender specific tailoring of interventions are warranted. Although PA levels seem stable over time among adolescent, this is by far the age-group with the largest potential for improving PA levels. Thus, we believe that the best buy for future public health would be to target children and adolescent independent of gender and age. This approach is supported by longitudinal data from the Gateshead Millennium Study [34] where there was no evidence that declines in PA began during adolescence, or that adolescent declines in PA were greater than the declines during childhood. Identifying temporal trends in PA during different day types and day segments could provide even more detailed information on when such efforts may have potential for success. We observed that temporal changes in general have been more pronounced during weekdays and school hours than during weekend days. Moreover, data from a large multi-country accelerometer-measured physical activity dataset showed that children accumulated significantly more MVPA on weekdays versus weekend days [35]. This suggest a potential for increasing PA levels related to segments such as active transport to/from school, PA during school hours and weekday leisure activity. Such strategies are supported by evidence from a large systematic review revealing that active school travellers were more physically active and that active school transport interventions lead to increase in PA [36], and likewise a recent school-based cluster randomized PA intervention showed to be effective in curbing a decline in PA levels among adolescents [37].

### Strengths and limitations

A major strength of this study is the systematic, repeated monitoring of large population-based samples of both children and adolescents. In order to monitor health habits, and to evaluate the effect of various public health actions, repeated surveys in population-based samples are a requisite. Objective, valid and reliable measurement methods have been used, ensuring high quality data, and the systematic approach carried out since 2005 is unique in the national and global context. Along with geographical representativeness, both participation (n) and participation rates (%) need to be large enough to ensure nationally representative data and to ensure that samples included are comparable to the general population. As 4582 of the 14,082 invited chose not to participate (32.5%), we cannot rule out selection bias as we do not have access to data allowing a formal comparison of our analytical samples to the general population. However, in PANCS2 we linked participants to registry data on parental education and a crude comparison of these data with aggregate 2011 data on education levels were comparable to the proportions in the general population. Similar explorations are difficult for PANCS1 and PANCS3 due to missing data on parental education. However, the participation rates in PANCS3 were very similar to the participation rates in PANCS2, and we have no strong reason to believe that non-participants should substantially differ between 2011 and 2018.

Participation rates are somewhat higher in PANCS1 compared to PANCS2 and 3, and thus the comparability across time points should be interpreted with this in mind. We must, however, emphasize that participation rates are conservatively calculated, as we do not know how many of the non-responding children and adolescents received the invitation to participate (e.g. they may have been absent from school). Nonetheless, studies with device measured PA at the national level is still very rare and the relatively high participation rates obtained in all three age groups and the geographical representativeness indicates the results are generalisable.

The use of device-based measures of PA has many advantages, but also some weaknesses. Because the participants wore the monitors on their hip, and because they are not waterproof, it is unavoidable that PA intensity due to upper body movements (e.g. strength training), load carrying activities (e.g. carrying a backpack), other activities with little vertical hip movement (e.g. cycling), and water-based activities is underestimated. We cannot rule out that a shift towards more engagement in the abovementioned activities could potentially influence results. Lastly, because the ActiGraph model used in PANCS1 uses a different accelerometer than the models used in PANCS2 and 3, results should be interpreted with some caution. We did however correct for the inter-model differences in CPM output to account for this [38].

### Public health implications

To reach WHO’s goal of a 10% relative reduction in physical inactivity between 2010 and 2025, estimated from the 2011 survey data suggest that 6-, 9- and 15-year-olds (boys and girls combined) on group level need to increase MVPA by a modest 2–3 min/d (14–21 min/week) in average. Due to the decline in MVPA between 2011 and 2018 among 9-year-olds, this specific group is estimated to increase their time spent in MVPA by 5–6 min/d (35–42 min/week) in order to reach that target, and even more to reach the 2030 goal of a 15% reduction. Furthermore, the finding of decreased PA levels and lower proportion of sufficiently active individuals among the least active quintile should be of particular public health concern, as these children already are among those who would benefit the most from increased PA. Based on the present finding it seems apparent that we will not reach the WHO goal of reducing physical inactivity without a national strategy with higher engagement across multiple sectors and stakeholders.

## Conclusion

Overall, PA levels have been stable between 2005, 2011 and 2018 in Norwegian youth. However, the declining PA level among 9-year-old boys and the low proportion of 15-year-olds sufficiently active is concerning. Present findings indicate that efforts to increase PA levels among children and adolescents have been insufficient. To evaluate the effect of, and plan for new, PA promoting strategies, it is important to ensure more frequent, systematic, device-based monitoring of population-levels of PA.

## Supplementary Information

**Additional file 1. **Proportions of the analytical sample (*n* = 8186) that were boys and girls in PANCS1, 2 and 3 by age group.

**Additional file 2.** Inclusion criteria for secondary analyses of temporal changes in PA between 2005, 2011 and 2018.

**Additional file 3.** MVPA and proportion meeting PA guideline for the most and least active.

**Additional file 4.** Temporal changes among the most and least active.

## Data Availability

The datasets during and/or analysed during the current study are available from the corresponding author on reasonable request.
