# Supplementary material for: Temporal trends in physical activity levels across more than a decade – a national physical activity surveillance system among Norwegian children and adolescents
Source: Int J Behav Nutr Phys Act. 2021 Apr 26;18:55. doi: 10.1186/s12966-021-01120-z (PMC8074468; doi:10.1186/s12966-021-01120-z)
Supplement: Supplementary file 1 — Additional file 1. Proportions of the analytical sample (n = 8186) that were boys and girls in PANCS1, 2 and 3 by age group. [file 12966_2021_1120_MOESM1_ESM.docx]

**Additional file 1. Proportions of the analytical sample (n=8,186) that were boys and girls in PANCS1, 2 and 3 by age group**

|  | **Boys** | **Girls** |
| --- | --- | --- |
| **6-y-olds** |  |  |
| 2011 (PANCS2) | n=494 (49.2%) | n=510 (50.8%) |
| 2018 (PANCS3) | n=411 (49.1%) | n=427 (50.9%) |
| **9-y-olds** |  |  |
| 2005 (PANCS1) | n=599 (53.3%) | n=524 (46.7%) |
| 2011 (PANCS2) | n=652 (48.5%) | n=692 (51.5%) |
| 2018 (PANCS3) | n=607 (49.8%) | n=613 (50.2) |
| **15-y-olds** |  |  |
| 2005 (PANCS1) | n=340 (48.6%) | n=360 (51.4%) |
| 2011 (PANCS2) | n=480 (49.5%) | n=489 (50.5%) |
| 2018 (PANCS3) | n=491 (49.7%) | n=497 (50.3%) |
