# Supplementary material for: Temporal trends in physical activity levels across more than a decade – a national physical activity surveillance system among Norwegian children and adolescents
Source: Int J Behav Nutr Phys Act. 2021 Apr 26;18:55. doi: 10.1186/s12966-021-01120-z (PMC8074468; doi:10.1186/s12966-021-01120-z)
Supplement: Supplementary file 2 — Additional file 2. Inclusion criteria for secondary analyses of temporal changes in PA between 2005, 2011 and 2018. [file 12966_2021_1120_MOESM2_ESM.docx]

**Additional file 2. Inclusion criteria for secondary analyses of temporal changes in PA between 2005, 2011 and 2018**

|  |  | **Day/segment** | **Valid day/segment** | **Included if # of valid days/segments** |
| --- | --- | --- | --- | --- |
| Weekdays | | 6:00-00:00 | ≥480 minutes | ≥2-5 |
| Weekend days | | 6:00-00:00 | ≥480 minutes | ≥1-2 |
| School day segments | |  |  |  |
|  | Morning | 6:00-9:00 | ≥60 minutes | ≥2-5 |
|  | School | 9:00-13/14:00* | ≥180/240 minutes* | ≥2-5 |
|  | Afterschool | 13/14:00-16:00* | ≥120/≥60 minutes* | ≥2-5 |
|  | Afternoon | 16:00-00:00 | ≥180 minutes | ≥2-5 |

*13:00/≥180 for 6- and 9-y-olds, 14:00/≥240 for 15-y-olds.
