# Supplementary material for: Temporal trends in physical activity levels across more than a decade – a national physical activity surveillance system among Norwegian children and adolescents
Source: Int J Behav Nutr Phys Act. 2021 Apr 26;18:55. doi: 10.1186/s12966-021-01120-z (PMC8074468; doi:10.1186/s12966-021-01120-z)
Supplement: Supplementary file 3 — Additional file 3. MVPA and proportion meeting PA guideline for the most and least active. [file 12966_2021_1120_MOESM3_ESM.docx]

**Additional file 3. Mean (95% CI) MVPA minutes per day and overall and proportion meeting PA guideline (≥60 min/d of MVPA) for the 20% most and least active in 2005, 2011 and 2018 by age group and sex (6 year olds did not participate in PANCS1 (2005)).**

|  | **MVPA (minutes per day)** | | | | | |  | **Proportion meeting PA guideline (≥60 min/d of MVPA)*** | | | | | |
| --- | --- | --- | --- | --- | --- | --- | --- | --- | --- | --- | --- | --- | --- |
|  | **2005** | **n** | **2011** | **n** | **2018** | **n** |  | **2005** | **n** | **2011** | **n** | **2018** | **n** |
| **20 % least active** |  |  |  |  |  |  |  |  |  |  |  |  |  |
| Boys 6 | - |  | 66.1 (64.1, 68.1) | 99 | 61.6 (59.6, 63.7) | 83 |  | - |  | 77% | 99 | 55% | 83 |
| Girls 6 | - |  | 55.7 (54.1, 57.4) | 102 | 52.7 (51.1, 54.4) | 85 |  | - |  | 38% | 102 | 20% | 85 |
| Boys 9 | 57.7 (55.7, 59.8) | 120 | 54.2 (52.2, 56.3) | 131 | 51.3 (49.8, 52.8) | 122 |  | 65% | 120 | 46% | 131 | 11% | 122 |
| Girls 9 | 45.3 (43.2, 47.3) | 105 | 44.0 (42.5, 45.5) | 139 | 43.8 (42.8, 44.9) | 123 |  | 0% | 105 | 0% | 139 | 0% | 139 |
| Boys 15 | 34.7 (32.9, 36.6) | 69 | 34.4 (32.5, 36.2) | 96 | 31.1 (29.3, 32.9) | 99 |  | 0% | 69 | 0% | 96 | 0% | 96 |
| Girls 15 | 31.0 (29.5, 32.5) | 73 | 31.3 (30.0, 32.6) | 98 | 32.3 (30.9, 33.6) | 100 |  | 0% | 73 | 0% | 98 | 0% | 100 |
| **20% most active** |  |  |  |  |  |  |  |  |  |  |  |  |  |
| Boys 6 | - |  | 141.2 (138.2, 144.2) | 98 | 132.8 (129.9, 135.7) | 82 |  | - |  | 100% | 98 | 100% | 82 |
| Girls 6 | - |  | 114.4 (112.2, 116.7) | 102 | 107.7 (104.7, 110.8) | 85 |  | - |  | 100% | 102 | 100% | 85 |
| Boys 9 | 144.4 (140.8, 148.0) | 119 | 131.7 (129.5, 133.8) | 130 | 120.5 (117.9, 123.2) | 121 |  | 100% | 119 | 100% | 130 | 100% | 121 |
| Girls 9 | 112.0 (109.1, 114.9) | 105 | 105.2 (102.8, 107.6) | 138 | 102.4 (100.0, 104.8) | 122 |  | 100% | 105 | 100% | 138 | 100% | 122 |
| Boys 15 | 111.5 (106.5, 116.5) | 68 | 105.9 (102.7, 109.0) | 96 | 106.4 (103.0, 109.8) | 98 |  | 100% | 68 | 100% | 96 | 100% | 98 |
| Girls 15 | 98.6 (95.7, 101.6) | 72 | 89.9 (87.1, 92.7) | 97 | 94.7 (90.2, 99.1) | 99 |  | 100% | 72 | 100% | 97 | 100% | 99 |

*Crude (i.e. not proportions adjusted for differences in age, daylight and wear time in logit model – the logit model naturally does not handle a dependant variable that does not vary (0 or 100% adherence to the guideline)).
