# Supplementary material for: Temporal trends in physical activity levels across more than a decade – a national physical activity surveillance system among Norwegian children and adolescents
Source: Int J Behav Nutr Phys Act. 2021 Apr 26;18:55. doi: 10.1186/s12966-021-01120-z (PMC8074468; doi:10.1186/s12966-021-01120-z)
Supplement: Supplementary file 4 — Additional file 4. Temporal changes among the most and least active. [file 12966_2021_1120_MOESM4_ESM.docx]

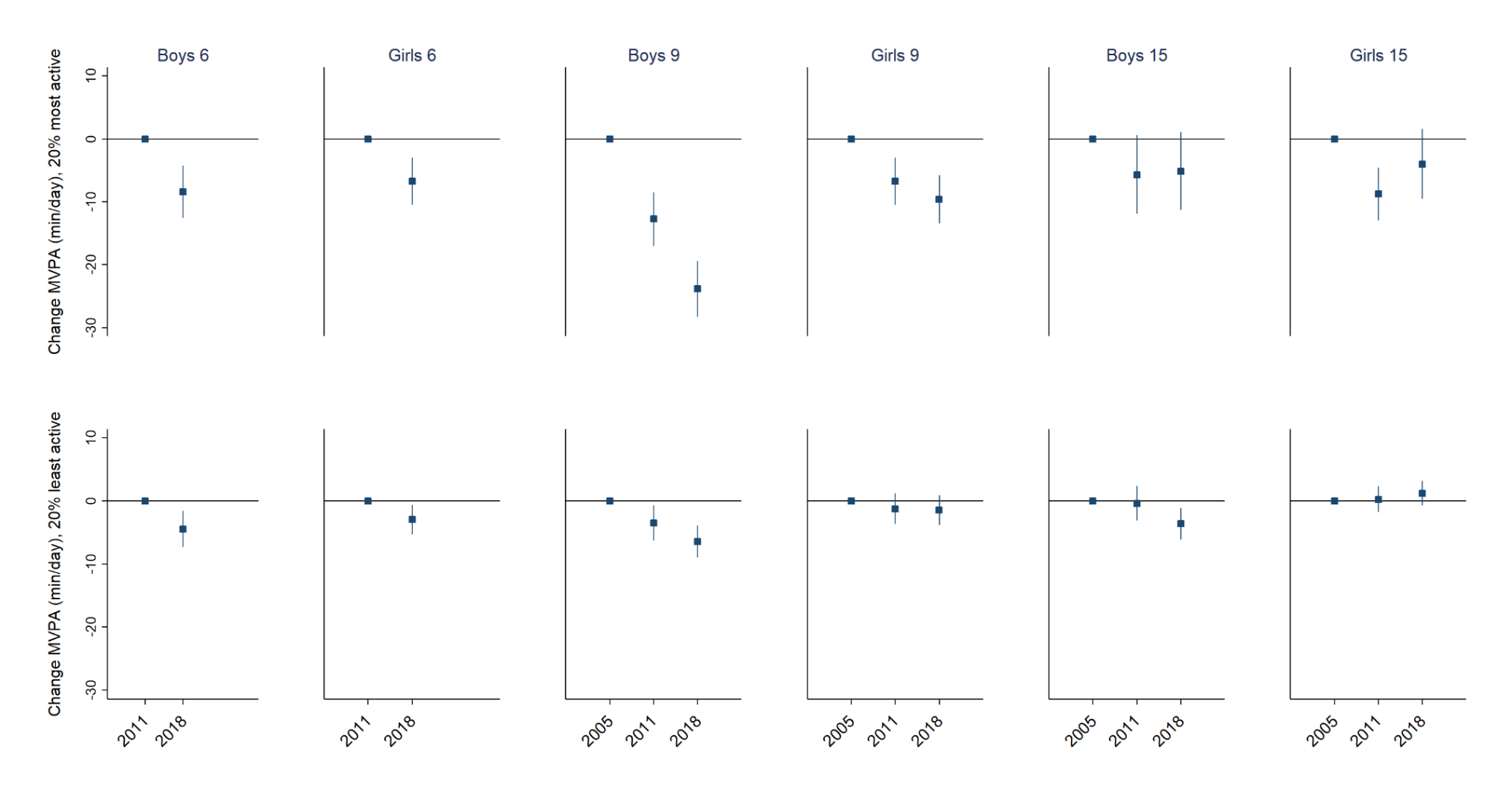
**Additional file 4. Temporal changes among the 20% most and least active 6-, 9- and 15-y-olds between cohorts (n ranges from 68 (15 y old boys) to 139 (9 y old girls** ))

MVPA; moderate-to-vigorous physical activity
